# Assessment of sustainable urban transport development based on entropy and unascertained measure

**DOI:** 10.1371/journal.pone.0186893

**Published:** 2017-10-30

**Authors:** Yancang Li, Jing Yang, Huawang Shi, Yijie Li

**Affiliations:** College of Civil Engineering, Hebei University of Engineering, Handan, China; Southwest University, CHINA

## Abstract

To find a more effective method for the assessment of sustainable urban transport development, the comprehensive assessment model of sustainable urban transport development was established based on the unascertained measure. On the basis of considering the factors influencing urban transport development, the comprehensive assessment indexes were selected, including urban economical development, transport demand, environment quality and energy consumption, and the assessment system of sustainable urban transport development was proposed. In view of different influencing factors of urban transport development, the index weight was calculated through the entropy weight coefficient method. Qualitative and quantitative analyses were conducted according to the actual condition. Then, the grade was obtained by using the credible degree recognition criterion from which the urban transport development level can be determined. Finally, a comprehensive assessment method for urban transport development was introduced. The application practice showed that the method can be used reasonably and effectively for the comprehensive assessment of urban transport development.

## Introduction

With the rapid development of the national economy and urbanization process, city size has been increasing rapidly, and transport demand and energy consumption have been soaring [1–4]. More serious urban transport pollution concerns have arisen, such as global warming, acid rain, smog pollution and urban haze. Urban transport pollution accounts for 80% of urban total pollution. Obviously, air pollution, noise pollution and hazardous wastes produced by urban transport have become the main sources of urban pollution [5–8]. The rapid growth of urban transport not only causes serious environmental pollution but also increases the energy consumption of urban transport. The energy consumption of urban transport also accounts for a high proportion of urban energy consumption. In the urban transportation system of the United States and Canada, transport fuel consumption accounts for approximately 60% and 30% of urban total consumption, respectively. However, with the increasing expansion and development of urban transport demand and urban roads, the proportion of energy consumption in the urban transport system will increase year by year. It will also introduce more serious challenges for energy reserves and supply in China. Thus, urban transport development should meet the growing urban transport demand and consider the negative impacts on environment and energy.

Based on this situation, the theory of sustainable urban transport development is of timely importance. Sustainable urban transport development meets the demand under the principle of fair and balanced development considering the urban environment bearing capacity and the energy consumption rate, ultimately achieving the goal of sustainable development. However, to measure whether the goal of sustainable development is realized in an urban transport system, an assessment index system should be established. Therefore, how to establish the evaluation index system has become the key problem of sustainable urban transport development research.

So far, many researchers have studied the assessment of sustainable development of urban transport, and many useful ideas have been proposed [9–21]. All these works have promoted the emergence of sustainable development of urban transport. However, the assessment index system for the sustainable development of urban transport is still not mature. Here, we established an evaluation index system for the sustainable development of urban transport based on aspects of urban economic development, transport demand, urban environment quality, and resource consumption. The entropy theory and the unascertained measure theory were employed to establish the comprehensive assessment model to identify the development degree of the urban transport system and then to guide and control sustainable development of the urban transport system.

## Methods

### Establishment of a comprehensive assessment index system

The selection of comprehensive assessment indexes and the treatment of the weight have a great influence on the assessment results. The connotations and characters of sustainable development of transportation are discussed. The design of the index system for evaluating the sustainable development of transportation should follow the scientific, objective, systematic, comparable and workable principles. Based on principles of scientific validity, systematization, normality and operability, a scientific and reasonable index system was established. Considering that urban economic development, transport demand, energy consumption and environmental pollution caused by urban transport development are the main factors influencing the sustainable development of urban transport, we established a comprehensive assessment indexes system that includes the coordinated development of urban transport and social economy, transport demand, environment quality and resource consumption.

The comprehensive assessment indexes system is shown as follow, and its details are shown in Table 1.

**Table 1:** Assessment system for the sustainable development of urban transport.

Target layer A	State layer B	Index layer C
Urban transport sustainable development A	Economic development B_1_	Per capita GDP C_1_
		Growth rate of GDP (%) C_2_
		Urban transport infrastructure investment proportion (%) C_3_
		Urban transport management facilities investment proportion (%) C_4_
	Transport demand B_2_	Road area ratio (%) C_5_
		Per capita road area (square metre) C_6_
		Urban road network density (km/sq km) C_7_
		Main road density (km/sq km) C_8_
	Urban environment quality B_3_	Motor vehicle tail gas passing rate (%) C_9_
		Air pollution saturation of a road transport (%) C_10_
		Over standard rate of section air quality (%) C_11_
		Annual average of main road noise (db) C_12_
	Resource consumption of urban transport B_4_	Consumption proportion of urban transport land (%) C_13_
		Resource consumption index of urban transport (%) C_14_

### Unascertained measure model

Suppose *x*_*i*_(*i* = 1,⋯,*n*) is the urban transport development status of the *i*^*th*^ year in a city. *X* = (*x*_1_,⋯,*x*_*n*_) is named as a universe. Assessment object *x*_*i*_(*x*_*i*_ ∈ *X*) has *m* evaluation indexes. *I* = (*I*_1_,⋯,*I*_*m*_) is an index set. *x*_*ij*_ is an observation value of assessment object *x*_*i*_ under index *I*_*j*_. *C*_*k*_(1 ≤ *k* ≤ *K*) is the *k*^*th*^ comment grade. In addition, *C*_*k*_ = (*c*_1_,⋯,*c*_*k*_) is a comment set. Suppose *A* is a standard matrix. Therefore, the standard matrix *A* can be shown:

$$A=\left[\begin{array}{cccc} a_{11} & a_{12} & \cdots & a_{1k}\\ a_{21} & a_{22} & \cdots & a_{2k}\\ \vdots & \vdots & \ddots & \vdots \\ a_{m1} & a_{m2} & \cdots & a_{mk} \end{array}\right]$$ (1)

where *a*_*jk*_ is a number under index *I*_*j*_(*j* = 1,⋯,*m*) and comment grade *C*_*k*_(*k* = 1,⋯,*K*). *a*_*jk*_ satisfies *a*_*ji*_ > *a*_*j*2_ > ⋯ > *a*_*jk*_ or *a*_*ji*_ < *a*_*j*2_ < ⋯ < *a*_*jk*_.

### Determination of single index unascertained measure

Suppose *x*_*ij*_ is the value of the *j*^*th*^ index for the *i*^*th*^ assessment object. *u*_*ijk*_ = *u*(*x*_*ij*_ ∈ *C*_*k*_) is the unascertained measure of *x*_*ij*_ for grade *C*_*k*_.

$${\left(u_{ijk}\right)}_{m\times k}=\left[\begin{array}{cccc} u_{i11} & u_{i12} & \cdots & u_{i1k}\\ u_{i21} & u_{i22} & \cdots & u_{i2k}\\ \vdots & \vdots & \ddots & \vdots \\ u_{im1} & u_{im2} & \cdots & u_{imk} \end{array}\right]$$ (2)

*x*_*ij*_ ∈ *C*_*k*_ denotes that *x*_*ij*_ is in the grade *C*_*k*_ conditions. Meanwhile, *u* should satisfy the principles of polarity and additivity.

Suppose that *a*_*ji*_ < *a*_*j*2_ < ⋯ < *a*_*jk*_, then

1. When *x*_*ij*_ ≺ *a*_*j*1_
  $$\{\begin{array}{c} u_{ij1}=1\\ u_{ij2}=u_{ij3}=\cdots u_{ijk}=0 \end{array}$$ (3)
2. When *x*_*ij*_ ≻ *a*_*j*1_
  $$\{\begin{array}{c} u_{ijk}=1\\ u_{ij1}=u_{ij2}=\cdots u_{ijk-1}=0 \end{array}$$ (4)
3. When *a*_*j*k_ < *x*_*ij*_ < ⋯ < *a*_*jk*+1_
  $$\left\{ \begin{array}{l} u_{ijk}=\frac{a_{jk+1}-x_{ij}}{a_{jk+1}-a_{jk}}\\ u_{ijk+1}=\frac{x_{ij}-a_{jk}}{a_{jk+1}-a_{jk}}\\ u_{ij1}=u_{ij2}=\cdots =u_{jik-1}=0\\ u_{ijk+2}=u_{ijk+3}=\cdots =u_{ijk}=0 \end{array}\right.$$ (5)

### Determination of multi-index unascertained measure

#### Determination of weight coefficient for the assessment index

Entropy is a thermodynamic concept. It was introduced into information theory in 1948 by C. E. Shannon, who put forward the concept of information entropy to measure the level of system chaos or disorder [21–23]. Here, the Shannon information entropy, which is an objective and applicable method for the determination of weight value, was introduced into the comprehensive assessment.

Suppose that there are *m* assessment objects and *n* assessment indexes in a comprehensive assessment. The decision matrix *y*_*m*×*n*_ can be obtained:

$$y_{m\times n}=\left[\begin{array}{cccc} y_{11} & y_{12} & \cdots & y_{1n}\\ y_{21} & y_{22} & \cdots & y_{2n}\\ \vdots & \vdots & \ddots & \vdots \\ y_{m1} & y_{m2} & \cdots & y_{mn} \end{array}\right]$$ (6)

In the above formula, *y*_*ij*_ denotes that the assessment value of the assessment object *x*_*i*_ is the obtained assessment under assessment index *I*_*j*_.

After the decision matrix was obtained, normalization processing was needed. The normalization processing method is as follows:

1. For the profitability indexes:
  $$y_{ij}^{'}=\frac{y_{ij}-\min y_{j}}{\max y_{j}-\min y_{j}}$$ (7)
2. For the cost indexes:
  $$y_{ij}^{'}=\frac{\max y_{j}-y_{ij}}{\max y_{j}-\min y_{j}}$$ (8)

Here, the weights of the indexes were calculated using the entropy weight theory and the credible degree criteria.

In formulas (7) and (8), max *y*_*j*_ and min *y*_*j*_ denote, respectively, the maximum value and minimum value of assessment index *I*_*j*_.

Therefore, the degree of importance for assessment index *y*_*ij*_ under assessment index *I*_*j*_ can be expressed as *d*_*ij*_ assessment:

$$d_{ij}=\frac{y_{ij}^{\prime }}{\sum_{i=1}^{m}y_{ij}^{\prime }}$$ (9)

The entropy value of assessment index *I*_*j*_ is:

$$H_{j}=-k\sum_{i=1}^{m}d_{ij}\ln d_{ij}$$ (10)

where $k=\frac{1}{\ln m}$.

The entropy weight value of the index *I*_*j*_ is:

$$w(I_{j})=\frac{1-H_{j}}{\sum_{j=1}^{n}\left(1-H_{j}\right)}$$ (11)

### Determination of multi-index unascertained measure

Suppose *u*_*ik*_(*x*_*i*_ ∈ *C*_*k*_) is the multi-index unascertained measure, and it denotes the degree of assessment object *x*_*i*_ for grade *C*_*k*_; then

$$u_{ik}=\sum_{j=1}^{m}w(I_{j})u_{ijk},(1<i<n,1<k<K)$$ (12)

Then, the multi-index unascertained measure matrix can be obtained.

$${\left(u_{ik}\right)}_{n\times k}=\left[\begin{array}{cccc} u_{11} & u_{12} & \cdots & u_{1k}\\ u_{21} & u_{22} & \cdots & u_{2k}\\ \vdots & \vdots & \ddots & \vdots \\ u_{n1} & u_{n2} & \cdots & u_{nk} \end{array}\right]$$ (13)

### Credible degree recognition criterion and sort

The criterion of the degree of confidence can avoid the occurrence of unreasonable classification such as fuzzy synthetic assessment. In addition, it is indicative of good reliability, error capability, and stable performance. Because the grade *C*_*k*_ is orderly, the credible degree recognition criterion is used. The credible degree is usually selected as *λ*(0.5 < *λ* < 1); generally, *λ* = 0.6 or *λ* = 0.7 [24].

Suppose:

$$k_{i}=\min\left[k\;:\;\sum_{i=1}^{k}u_{ik}\geq \lambda ,1≺k≺K\right]$$ (14)

*k*_*i*_ indicates that assessment object *x*_*i*_ belongs to assessment grade *C*_*k*_.

The score criterion method is applied to the order for assessment object *x*_*i*_:

$$q(x_{i})=\sum_{k=1}^{K}n_{k}u_{ik}$$ (15)

where *n*_*k*_ denotes the score of the grade *C*_*k*_. *q*(*x*_*i*_) is the degree of uncertainty of the evaluation scheme. *Q* = {*Q*_1_, *Q*_2_, ⋯, *Q*_*n*_} is the vector of the degree of uncertainty; according to its size, the superior ranking can be finished.

## Results and discussion

To verify the effectiveness of the model proposed here, the sustainable development of urban transport in a city over six years was chosen as an example for the sustainable development assessment. The assessment indexes were selected from the aspects of urban economic development, transport demand, environment quality and resource consumption. The comprehensive assessment results were analysed. The assessment index grade standard of sustainable development of urban transport was established, as shown in Table 2, based on the *Evaluation index system of city road transport management* [25]. Values of the indexes of the sustainable development of urban transport from 2007 to 2012 in the city are shown in Table 3 where *C*_1_, *C*_2_, ⋯, *C*_*n*_ *n* = {1 2, ⋯, 14} are all the indexes shown in Table 1 and given in [25].

**Table 2:** Grading standard for the sustainable development of urban transport.

Assessment index		Grade divide				
		Ⅴ	Ⅳ	Ⅲ	Ⅱ	Ⅰ
B_1_	C_1_	<0.7	0.7–3	3–6	6–15	≥15
	C_2_	<3	3–5	5–8	8–10	≥10
	C_3_	<1.5	1.5–2	2–2.5	2.5–3	≥3
	C_4_	<1.5	1.5–2	2–2.5	2.5–3	≥3
B_2≥_	C_5_	<7	7–9	9–11	11–13	≥13
	C_6_	<4	4–6	6–8	8–11	≥11
	C_7_	1–4	4–5	5–6	6–7	≥7
	C_8_	1–2.5	2.5–3	3–3.5	3.5–4	≥4
B_3_	C_9_	<80	80–85	85–90	90–95	≥95
	C_10_	>0.9	0.75–0.9	0.6–0.75	0.4–0.6	<0.4
	C_11_	>30	20–30	15–20	10–15	<10
	C_12_	>75	70–75	65–70	60–65	<60
B_4_	C_13_	>6	4.5–6	3–4.5	2–3	<2
	C_14_	>0.8	0.6–0.8	0.4–0.6	0.2–0.4	<0.2

**Table 3:** The index value for the sustainable development of urban transport.

Index	2007	2008	2009	2010	2011	2012
C_1_	0.6	0.62	0.68	0.74	0.84	0.86
C_2_	14.5	9.1	10.2	10.3	8.1	7.7
C_3_	0.12	0.13	0.09	0.072	0.8	0.75
C_4_	0.08	0.07	0.1	0.92	0.8	0.82
C_5_	8.2	8.0	7.82	7.85	8.0	7.88
C_6_	4.60	5.62	5.73	8.45	8.6	8.8
C_7_	5.2	4.94	4.83	5.6	5.8	6.2
C_8_	1.2	1.42	1.6	1.54	2.1	2.3
C_9_	86.2	87.3	88	88	88.2	88.4
C_10_	1.42	1.34	1.28	1.26	1.0	0.98
C_11_	34	32	30	29	31	28
C_12_	66.4	67	68.3	69.1	69.4	69.6
C_13_	3.4	4.2	3.22	4.8	5.4	6.2
C_14_	0.8	0.82	0.75	0.74	0.65	0.56

The data of *C*_1_, *C*_2_, ⋯, *C*_*n*_ *n* = {1 2, ⋯, 14} are from the statistical yearbook of the city.

First, normalization process was finished by using Eq (7) and Eq (8). Then, the multi-index unascertained measure was determined. The information entropy theory and the credible degree recognition criterion were used. Finally, the comprehensive assessment results were obtained. The comprehensive assessment results are shown in Table 4.

**Table 4:** The result of the comprehensive assessment.

Year	2007	2008	2009	2010	2011	2012
Development score	3.892	4.265	4.049	3.993	4.106	4.075
Development grade	Ⅱ	Ⅱ	Ⅱ	Ⅲ	Ⅲ	Ⅲ

There are strengths and weaknesses to these tables and findings in relation to the assessment of sustainable transport based on the measurement adopted. From the comprehensive assessment results above, we realize that the sustainable development of transport in this city demonstrated better performance during five years. The order of the development level from high to low was 2008, 2011, 2*0*12, 2009, 2010, and 2007. The development level from 2007 to 2009 was good, and it was in general status from 2010–2012. From that, we also know that the sustainable development of urban transport started to slow down after 2009.

From the table, we can also determine the contribution rate of each index. The changes in each index showed that the sustainable development was affected most by the per capita GDP and GDP growth rate in the economic development system. In the transport demand system, the index of the per capita road area had the greatest effect on development. The motor vehicle tail gas passing rate and the over standard rate of section air had a greater impact on development in the urban environment system. In the resource consumption system of the urban transport, the consumption proportion of urban transport land and resource consumption index of urban transport were both higher.

From the results above, we identify specific measures and methods to ensure the sustainable development of urban transport. Specifically, appropriate measures should be taken to alleviate the urban environment pressure and to reduce the excessive consumption of resources by urban transport in urban transport development. To alleviate urban environment pressure, renewable and green energy such as ethanol gasoline should be considered for automotive use. In addition, the stereoscopic transport space should be utilized fully to reduce urban transport land consumption.

## Conclusion

Along with the rapid development of urban transport, the sustainable development assessment of urban transport becomes more and more important. Because usual control methods result in subjectivity defects, the comprehensive assessment model for the sustainable development of urban transport was established based on the information entropy theory and the unascertained measure. In the model, the weight of each index was determined using the information entropy coefficient method. The credible degree recognition criterion was applied to analyze the comprehensive assessment results. Then, the model was introduced in the assessment of sustainable development of urban transport. By applying the model in practice, the feasibility and effectiveness of the established model were verified. At the same time, the urban transport development tendency and the main factors influencing the sustainable development of urban transport were determined. All these factors are what we shall direct energy to in the future. The analysis of the comprehensive assessment results can guide the city to take the relevant improved and managerial measures to ensure sustainable development of urban transport. This study provides an effective method for the comprehensive assessment of urban transport development. In subsequent work, we will make further efforts to focus on establishing an index system and the calculating the weights of the factors.

## Supporting information

S1 Fig The development and running statement of urban transport. (TIF)

S1 Table Assessment system for urban transport sustainable development. (PDF)

S2 Table Grading standard for urban transport of sustainable development. (PDF)

S3 Table The index value for urban transport of sustainable development. (PDF)

S4 Table The result of the comprehensive assessment. (PDF)
