# Supplementary figures and images for: Assessment of sustainable urban transport development based on entropy and unascertained measure
Source: PLoS One. 2017 Oct 30;12(10):e0186893. doi: 10.1371/journal.pone.0186893 (PMC5662088; doi:10.1371/journal.pone.0186893)

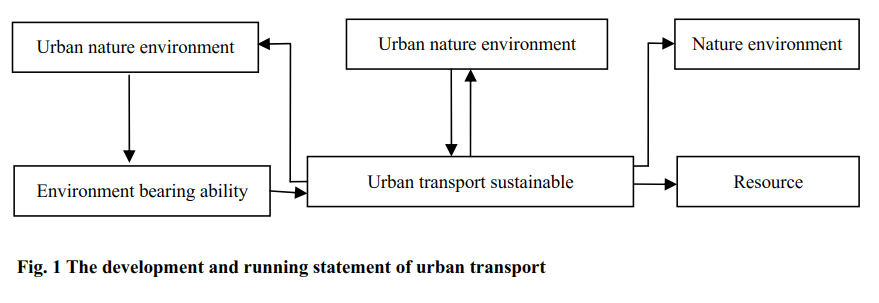

Supplement: S1 Fig — (TIF) [file pone.0186893.s001.tif]
