# Supplementary material for: Assessment of sustainable urban transport development based on entropy and unascertained measure
Source: PLoS One. 2017 Oct 30;12(10):e0186893. doi: 10.1371/journal.pone.0186893 (PMC5662088; doi:10.1371/journal.pone.0186893)
Supplement: S1 Table — (PDF) [file pone.0186893.s002.pdf]

Table 1 Assessment system for urban transport sustainable development

Target layer A	State layer B	Index layer C
Urban transport sustainable development A	Economic development B_1	Per capita GDP C_1
		Growth rate of GDP(%)C_2
		Urban transport infrastructure investment proportion(%)C_3
		Urban transport management facilities investment proportion(%)C_4
	Transport demand B_2	Road area ratio(%)C_5
		Per capita road area(Square meter)C_6
		Urban road network density(Km/sq km)C_7
		Main road density(Km/sq km)C_8
	Urban environment quality B_3	Motor vehicle tail gas passing rate(%)C_9
		Air pollution saturation of road transport(%)C_{10}
		Over standard rate of section air quality(%)C_{11}
		Annual average of main road noise (db)C_{12}
	Resource consumption of urban transport B_4	Consumption proportion of urban transport land(%)C_{13}
		Resource consumption index of urban transport(%)C_{14}
