# Supplementary material for: Assessment of sustainable urban transport development based on entropy and unascertained measure
Source: PLoS One. 2017 Oct 30;12(10):e0186893. doi: 10.1371/journal.pone.0186893 (PMC5662088; doi:10.1371/journal.pone.0186893)
Supplement: S2 Table — (PDF) [file pone.0186893.s003.pdf]

Table 2 Grading standard for urban transport of sustainable development

Assessment index		Grade divide				
		V	IV	III	II	I
B_1	C_1	<0.7	0.7-3	3-6	6-15	≥15
	C_2	<3	3-5	5-8	8-10	≥10
	C_3	<1.5	1.5-2	2-2.5	2.5-3	≥3
	C_4	<1.5	1.5-2	2-2.5	2.5-3	≥3
$B_{2\geq}$	C_5	<7	7-9	9-11	11-13	≥13
	C_6	<4	4-6	6-8	8-11	≥11
	C_7	1-4	4-5	5-6	6-7	≥7
	C_8	1-2.5	2.5-3	3-3.5	3.5-4	≥4
B_3	C_9	<80	80-85	85-90	90-95	≥95
	C_{10}	>0.9	0.75-0.9	0.6-0.75	0.4-0.6	<0.4
	C_{11}	>30	20-30	15-20	10-15	<10
	C_{12}	>75	70-75	65-70	60-65	<60
B_4	C_{13}	>6	4.5-6	3-4.5	2-3	<2
	C_{14}	>0.8	0.6-0.8	0.4-0.6	0.2-0.4	<0.2
