# Supplementary material for: Assessment of sustainable urban transport development based on entropy and unascertained measure
Source: PLoS One. 2017 Oct 30;12(10):e0186893. doi: 10.1371/journal.pone.0186893 (PMC5662088; doi:10.1371/journal.pone.0186893)
Supplement: S3 Table — (PDF) [file pone.0186893.s004.pdf]

Table 3 The index value for urban transport of sustainable development

Index	2007	2008	2009	2010	2011	2012
C_1	0.6	0.62	0.68	0.74	0.84	0.86
C_2	14.5	9.1	10.2	10.3	8.1	7.7
C_3	0.12	0.13	0.09	0.072	0.8	0.75
C_4	0.08	0.07	0.1	0.92	0.8	0.82
C_5	8.2	8.0	7.82	7.85	8.0	7.88
C_6	4.60	5.62	5.73	8.45	8.6	8.8
C_7	5.2	4.94	4.83	5.6	5.8	6.2
C_8	1.2	1.42	1.6	1.54	2.1	2.3
C_9	86.2	87.3	88	88	88.2	88.4
C_{10}	1.42	1.34	1.28	1.26	1.0	0.98
C_{11}	34	32	30	29	31	28
C_{12}	66.4	67	68.3	69.1	69.4	69.6
C_{13}	3.4	4.2	3.22	4.8	5.4	6.2
C_{14}	0.8	0.82	0.75	0.74	0.65	0.56

Table 4 The result of the comprehensive assessment

Year	2007	2008	2009	2010	2011	2012
Development score	3.892	4.265	4.049	3.993	4.106	4.075
Development grade	II	II	II	III	III	III
