# Supplementary material for: Assessment of sustainable urban transport development based on entropy and unascertained measure
Source: PLoS One. 2017 Oct 30;12(10):e0186893. doi: 10.1371/journal.pone.0186893 (PMC5662088; doi:10.1371/journal.pone.0186893)
Supplement: S4 Table — (PDF) [file pone.0186893.s005.pdf]

Table 4 The result of the comprehensive assessment

Year	2007	2008	2009	2010	2011	2012
Development score	3.892	4.265	4.049	3.993	4.106	4.075
Development grade	II	II	II	III	III	III
